# Changing Trends in the Incidence and Clinical Features of *Pneumocystis jirovecii* Pneumonia in Non-HIV Patients before and during the COVID-19 Era and Risk Factors for Mortality between 2016 and 2022

**DOI:** 10.3390/life13061335

**Published:** 2023-06-07

**Authors:** Jin Suk Kang

**Affiliations:** Division of Infectious Diseases, Department of Internal Medicine, Inje University College of Medicine, Inje University Busan Paik Hospital, 75, Bokji-ro, Busanjin-gu, Busan 47392, Republic of Korea; gmlesnddl06@naver.com; Tel.: +82-51-890-6986

**Keywords:** *Pneumocystis jirovecii* pneumonia, COVID-19, pandemic, incidence, mortality, risk factor

## Abstract

Fungal superinfections have been reported in patients with coronavirus disease 2019 (COVID-19). We analyzed the incidence and clinical characteristics of *Pneumocystis jirovecii* pneumonia (PCP) in non-human immunodeficiency virus patients at a tertiary hospital between 2016 and 2022 to evaluate the impact of the COVID-19 pandemic on PCP. The study period was divided into pre-COVID-19 and COVID-19 eras based on the pandemic declaration by the World Health Organization. Among the 113 patients included, the incidence of PCP in the COVID-19 era (37/1000 patient-years) was significantly higher than that in the pre-COVID-19 era (13.1/1000 patient-years) (*p* < 0.001). Co-infection with invasive pulmonary aspergillosis (IPA) also increased (2.4% vs. 18.3%, *p* = 0.013). Independent risk factors for PCP-related mortality were previous glucocorticoid use, hypoxemia, acute kidney injury, and IPA co-infection. Risk factors for IPA in patients with PCP included previous use of tyrosine kinase inhibitors, COVID-19 infection within 30 days, leukopenia, and intensive care unit admission. In the COVID-19 era, 12 (16.9%) patients with PCP had a history of COVID-19 infection within 90 days; however, infection was not associated with mortality. Active evaluation of patients with suspected PCP and assessment of IPA co-infection risk may help improve the outcomes of patients with PCP.

## 1. Introduction

Coronavirus disease 2019 (COVID-19), caused by the severe acute respiratory syndrome coronavirus 2 (SARS-CoV-2), has led to the emergence of various mutant strains [1,2,3]. In South Korea, after the first confirmed case in 2020, enhanced social distancing measures by the Korean government helped control the spread of the virus [4,5,6,7]. However, the emergence of the Omicron SARS-CoV-2 variant in 2022 led to approximately 50% of the population being infected with COVID-19 [6,7]. As a result, there has been an increase in the number of patients recovering from COVID-19 infections [8,9]. Following COVID-19 infections, 5–30% of patients experience long COVID, characterized by various complications [10,11]. The duration of long COVID is reported to be 9 months for hospitalized patients and 4 months for non-hospitalized patients, and the rate of hospitalization for respiratory symptoms following acute COVID-19 infections has increased [12,13].

COVID-19 damages epithelial barriers and induces an abnormal immune response due to the dysregulation of the immune system [14,15]. Consequently, secondary pulmonary infections occur in hospitalized patients with COVID-19 pneumonia [16,17,18,19]. Fungal superinfections such as invasive pulmonary aspergillosis (IPA) have been reported, especially in patients who received immunosuppressive therapies or those with underlying conditions [17,19,20,21]. Recent studies have shown the detection of *Pneumocystis jirovecii* in respiratory specimens from critically ill patients with COVID-19 and case reports of co-infection with *P. jirovecii* pneumonia (PCP) in patients with severe COVID-19 [16,21,22,23,24,25]. Furthermore, several studies have reported cases of PCP occurring after COVID-19 recovery [23,26]. The time from onset of COVID-19 to onset of PCP varies from 8 to 180 days, and this infection can occur in patients without preexisting immunosuppressive conditions [23,26]. In those who are immunocompromised, PCP is a life-threatening opportunistic infection, and its incidence has increased with the use of immunosuppressive drugs for various diseases [27,28,29]. However, no studies have yet investigated the impact of the COVID-19 pandemic on the incidence, clinical characteristics, and outcomes of PCP. 

Herein, we analyzed the incidence and clinical characteristics of PCP in patients with non-human immunodeficiency virus (HIV) infection at a tertiary hospital before and during the COVID-19 pandemic. We also evaluated risk factors associated with mortality in patients with PCP between 2016 and 2022. 

## 2. Materials and Methods

### 2.1. Study Population and Design

We retrospectively analyzed the medical records of patients with PCP admitted to Inje University Busan Paik Hospital, Busan, South Korea, between January 2016 and December 2022. The study hospital was a university-affiliated tertiary hospital with 840 beds, four different intensive care units (ICUs), and one hematopoietic stem cell transplantation unit. The diagnosis of PCP was confirmed by positive polymerase chain reaction (PCR) testing of respiratory specimens such as sputum or bronchoalveolar lavage (BAL) from patients with clinical manifestations (fever, cough, sputum, dyspnea, or hypoxemia), and radiologic findings such as diffuse or patchy ground-glass opacification. Patients with positive *P. jirovecii* PCR results on respiratory samples who did not receive appropriate treatment for PCP because they were judged to be pulmonary colonies were excluded. Patients younger than 18 years of age and those with an HIV infection were also excluded from the study. 

To identify changes before and during the COVID-19 pandemic, since the use of immunosuppressive drugs for various diseases increased in the recent years compared to that in the past several decades [27,28], we included data from the last 7 years from 2016 to 2022, and evaluated the annual incidence of PCP. We assessed the impact of the COVID-19 pandemic on PCP by dividing our observations into two periods: the pre-COVID-19 era and the COVID-19 era, as defined by the World Health Organization (WHO) Declaration of the COVID-19 Pandemic (11 March 2020). We analyzed the prevalence, incidence, and clinical features of PCP in the hospitalized patients during each period. We evaluated risks factors contributing to mortality due to PCP during those 7 years, including the effects of recent COVID-19 infection, in the COVID-19 era. Patients whose treatment outcomes were unknown because they were transferred to another hospital were excluded from mortality risk factor analysis. 

### 2.2. Data Collection and Definitions

Demographic and clinical features such as age, sex, underlying diseases, Charlson comorbidity index (CCI) score, immunosuppressive treatment prior to the onset of PCP, 90 day history of COVID-19 infection, presentation with shock, co-infection with cytomegalovirus (CMV) and IPA during PCP treatment, ICU admission, and in-hospital mortality, including death due to PCP and underlying disease progression, were obtained from patients’ medical records. According to the hospital’s infection control policy to prevent COVID-19 transmission, Inje University Busan Paik Hospital conducted COVID-19 PCR on all patients upon admission and assessed for a history of COVID-19 infection within the past 90 days. A history of COVID-19 infection was defined as PCR diagnosis or COVID-19 antigen tests conducted at hospitals and public health centers. Patients who were self-diagnosed by COVID-19 antigen tests were excluded. According to the European Organization for Research and Treatment of Cancer and the Mycoses Study Group Education and Research Consortium (EORTC/MSGERC) [30], IPA infection was defined as one that met the criteria for probable or possible IPA. Patients with a history of COVID-19, even without immunocompromised host factors, were diagnosed with IPA if they had radiological and microbial findings [31]. Ten patients with probable IPA and two with possible IPA were identified according to the EORTC/MSGERC criteria, and two patients were diagnosed with a history of COVID-19 infection within 30 days [30,31]. All patients with IPA included in this study received mold-active antifungal treatment. CMV infection was defined as a positive PCR or culture in BAL fluid or CMV DNA ≥500 copies/mL in serum, and as receiving effective antiviral therapy with clinical manifestations [32,33]. Glucocorticoid use was defined as its administration for ≥2 weeks in the past 60 days prior to PCP diagnosis [30]. Glucocorticoids were converted to prednisolone-equivalent doses. Moderate-to-severe PCP was defined as a room air PaO_2_ < 70 mmHg, an alveolar–arterial oxygen gradient ≥ 35 mmHg, and/or evidence of hypoxemia. Shock was defined as sepsis with persisting hypotension and requiring vasopressor therapy needed to maintain mean arterial pressure at ≥65 mmHg despite adequate fluid resuscitation [34]. Acute kidney injury was defined as an increase in serum creatinine by ≥0.3 mg/dL within 48 h, an increase in serum creatinine to ≥1.5 times the baseline, which is known or presumed to have occurred within the prior 7 days, or a urine volume of <0.5 mL/kg/h for 6 h [35]. 

### 2.3. Statistical Analysis

All statistical analyses were performed using IBM SPSS Statistics (version 27.0; IBM Corp., Armonk, NY, USA). Categorical variables were described using frequencies and percentiles, and continuous variables were summarized as medians and interquartile ranges (IQRs). Categorical variables were compared using Pearson’s χ^2^ tests or Fisher’s exact tests, whereas noncategorical variables were tested using Mann–Whitney U-tests. Multivariable analysis was performed for variables with a *p* value less than 0.05 in univariable analysis. The risk factors for disease-related mortality and IPA co-infection were analyzed using logistic regression analysis. Results with *p* values less than 0.05 were considered statistically significant.

## 3. Results

### 3.1. Prevalence, Incidence, and Clinical Features of P. jirovecii Pneumonia

**Figure 1:**
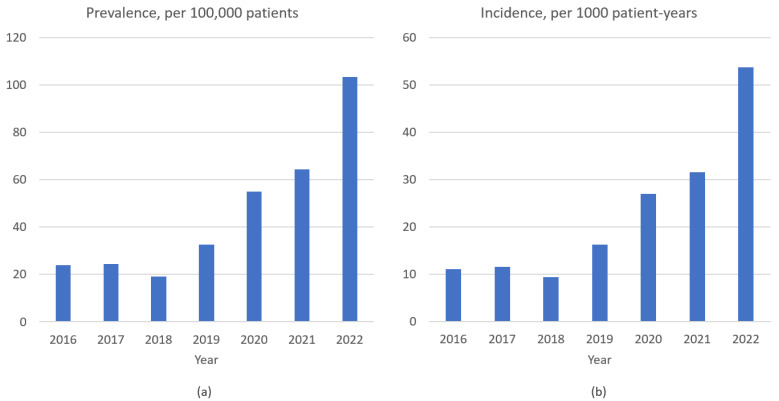
Annual prevalence (**a**) and incidence (**b**) of *P. jirovecii* pneumonia. The prevalence and incidence showed a gradual increasing trend after 2018. In 2022, the prevalence and incidence were 103.3/100,000 patients and 53.7/1000 patient-years, respectively.

During the study period, 250,959 patients over 5130 patient-years were admitted to the study hospital, of which 113 were patients with PCP. The overall prevalence and incidence of PCP were 45/100,000 patients and 22.1/1000 patient-years, respectively. The incidence of PCP during the COVID-19 era (37/1000 patient-years) was significantly higher than that during the pre-COVID-19 era (13.1/1000 patient-years) (Table 1). Additionally, the prevalence (103.3/100,000 patients) and incidence (53.7/1000 patient-years) of PCP further increased in 2022 (Figure 1). The median patient age was 69 years, with older patients with PCP in the COVID-19 era than before (Table 1). Lung cancer (15/43, 34.9%), lymphoma (7/21, 33.3%), and rheumatoid arthritis (15/23, 65.2%) were the most common solid cancer, hematological malignancy, and connective tissue disease, respectively. There were no significant differences between the pre- and the COVID-19 era in the underlying diseases or the use of immunosuppressive drugs, except glucocorticoids; more patients used ≥20 mg glucocorticoids in the pre-COVID-19 era. PCP prophylaxis, shock, and initial hypoxemia were not different between the two periods.

**Figure 2:**
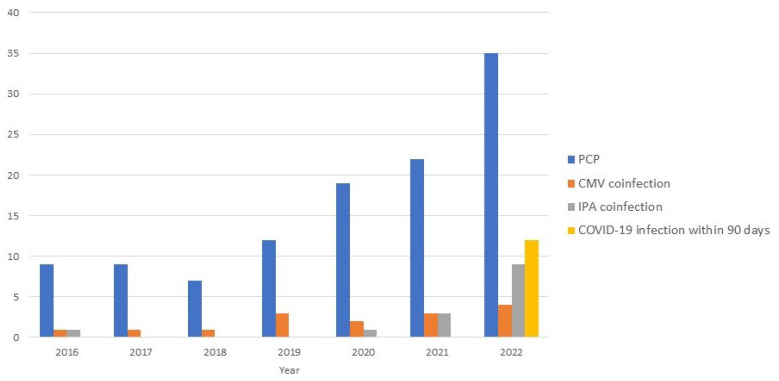
Number of cases of *P. jirovecii* pneumonia per year. IPA co-infection in patients with PCP was 14 (12.4%), of which 64.3% (9/14) were diagnosed in 2022. Twelve (10.6%) patients with PCP had a history of COVID-19 within 90 days. PCP, *P. jirovecii* pneumonia; CMV, cytomegalovirus; IPA, invasive pulmonary aspergillosis; COVID-19, coronavirus disease 2019.

Twelve (10.6%) patients with PCP had a history of COVID-19 within 90 days of hospitalization, all of whom were diagnosed with COVID-19 in 2022 (Table 1 and Figure 2). In 2022, 34.3% (12/35) of the patients with PCP had a history of COVID-19, and 25.7% (9/35) did not have pre-existing immunosuppressive conditions. The underlying diseases in patients with COVID-19 were solid tumors (5), hematological malignancies (4), and connective tissue disease (1). The median time from COVID-19 confirmation to PCP diagnosis was 63 days (IQR, 15–80 days; range, 1–90 days). Five (4.4%) patients with PCP had a history of COVID-19 infection within 30 days; three of these patients had severe COVID-19 pneumonia, one was identified with co-infection of COVID-19 at the time of PCP diagnosis, and one was diagnosed with PCP when his respiratory symptoms worsened again after his COVID-19 pneumonia improved. 

Fourteen (12.4%) patients presented with IPA co-infections, with a median time from PCP confirmation to diagnosis of IPA co-infection of 7 days (IQR, 3–20 days; range, 0–34 days). Nine (64.3%) patients with IPA co-infections were treated in the ICU. IPA co-infection increased significantly during the COVID-19 era (*p* = 0.013); no significant difference was observed for CMV co-infection (*p* = 0.807).

### 3.2. Outcome of P. jirovecii Pneumonia 

The PCP-related mortality rate was 44.6% (50/112), with no significant difference between the two periods (Table 1). One patient who was transferred to another hospital during the treatment and survival could not be confirmed. The characteristics according to PCP-related death are presented in Table 2 (N = 112). There were no significant differences in underlying disease or comorbidity between the two groups (Supplementary Table S1). Compared to the survivors, patients who did not survive had higher rates of previous glucocorticoid use, initial moderate-to-severe PCP, ICU admission, ventilator use, invasive pulmonary drainage, co-infection of IPA and CMV, acute kidney injury, and elevated values of lactate dehydrogenase (≥ 500 U/L) (*p* < 0.05).

### 3.3. Risk Factors Associated with PCP-Related Mortality 

We identified several independent risk factors for PCP-related mortality. These were: previous glucocorticoid use (adjusted odds ratio (aOR), 3.0; 95% confidence interval (CI), 1.1–8.1), initial moderate-to-severe PCP (aOR, 7.9; 95% CI, 1.8–34.3), acute kidney injury (aOR, 6.0; 95% CI, 2.1–16.8), and IPA co-infection (aOR, 7.2; 95% CI, 1.4–36.8) (Table 3). In multivariate analysis, CMV co-infection did not show a significant difference in PCP-related mortality (aOR, 3.3; 95% CI, 0.8–14.3). The risk factors for IPA co-infection in patients with PCP were the previous use of tyrosine kinase inhibitor, COVID-19 infection within the past 30 days, initial leukopenia (WBC <1000/μL), and ICU admission (*p* < 0.05) (Table 4). In the subgroup analysis for the COVID-19 era, COVID-19 infection within 90 days (aOR, 0.2; 95% CI, 0.0–1.6, *p* = 0.074) and within 30 days (aOR, 1.3; 95% CI, 0.2–9.6, *p* = 0.813) were not associated with mortality; moreover, in 2022, there was no significant difference according to COVID-19 within 90 days (aOR, 0.3; 95% CI, 0.1–1.6, *p* = 0.162) or within 30 days (aOR, 2.0; 95% CI, 0.2–16.4, *p* = 0.518).

## 4. Discussion

Our study revealed that the incidence of PCP increased during the COVID-19 pandemic, particularly in 2022, coincident with large-scale COVID-19 spread caused by Omicron mutations in South Korea. Risk factors for PCP-related mortality included previous glucocorticoid use, initial moderate-to-severe PCP, acute kidney injury, and IPA co-infection. Notably, IPA co-infection, a risk factor for mortality in PCP, was significantly increased compared to that in the pre-COVID-19 era. To the best of our knowledge, this is the first study to reveal the incidence and prognosis of PCP during the COVID-19 era. 

Although uncommon, bacterial and fungal infections occur after recovery from COVID-19 [16]. This viral infection causes an excessive increase in pro-inflammatory cytokines, leading to a compensatory anti-inflammatory response syndrome. If the immune response is excessive, long-term immune dysregulation may occur, increasing vulnerability to secondary infection and organ dysfunction, even after recovery from COVID-19 [10,14,15]. Immunocompromised patients are more susceptible to fungal diseases [13] and PCP is a lethal disease [28]. Recently, several studies have reported PCP superinfection in patients with severe COVID-19; however, there is no clear definition for distinguishing between infection and colonization [22]. Even after COVID-19 recovery, there have been case reports of PCP. The time from COVID-19 to PCP onset varies from 26 to 180 days, and some patients do not have preexisting PCP risk factors or experience severe COVID-19 infection [23,26]. However, there have been no detailed reports on the incidence and outcomes of PCP in the COVID-19 era when the number of patients recovering from COVID-19 is increasing. Park et al. reported that national non-pharmacological interventions, such as hand hygiene, social distancing, and isolation of symptomatic patients, reduced the incidence of PCP in kidney transplant patients during the COVID-19 era [36]. Conversely, Kim et al. reported that there was no change in PCP occurrence during the COVID-19 era, despite these governmental non-pharmacological interventions [37]. However, both studies investigated the incidence of PCP prior to June 2021 and, therefore, did not include the period from July 2021 to 2022, a period of rising COVID-19 cases in Korea [6,7]. Additionally, the findings from these previous reports were inconsistent with our observations, in which the incidence of PCP from January 2020 to June 2021 (26.4 patient-years) was higher than that in the pre-COVID-19 era. After the onset of the COVID-19 pandemic, the incidence of PCP increased, especially in 2022, when the Omicron SARS-CoV-2 variant became prevalent in Korea. Of the patients included in our study in 2022, two more were diagnosed with PCP, 171 and 169 days after COVID-19 infection, respectively; moreover, 40% (14/35) of patients with PCP had a history of COVID-19 infection, and 25.7% (9/35) had no pre-existing PCP risk factors. 

The mortality rate in non-HIV patients with PCP is high, between 35–50% [28]. Although the mortality rate reported in this study is similar to previous reports, the significant increase in PCP incidence may have led to an increase in the number of deaths. Initial hypoxemia was a risk factor for death, as shown in a previous study, in which a high alveolar–arterial oxygen gradient was associated with poor prognosis [32]. Chronic lung disease, initial blood urea nitrogen, lymphocyte count, lactate dehydrogenase, ventilator use, and CMV co-infection are known risk factors for death [32,38,39,40]. Although we did not find significant differences in these parameters in the present study, we identified acute kidney injury during treatment and previous glucocorticoid use as independent risk factors for PCP-related death. CMV co-infection, a known risk factor for death, was higher in non-survivors than in survivors; however, the difference was not significant. Liu et al. reported that CMV co-infection did not have a significant effect on mortality, but a high CMV DNA load in BAL was associated with mortality [41]. In the present study, we did not measure CMV DNA quantification in BAL fluid. 

IPA co-infection increased significantly during the COVID-19 pandemic. Zhong et al. reported that in-hospital mortality in non-HIV patients co-infected with PCP and IPA was 51% (26/51) [42]. In our study, PCP-related mortality was 71.4% (10/14), which was higher than that previously reported. Although a few studies have evaluated the effect of IPA co-infection on the outcome of PCP [39], our study confirms previous findings, suggesting that IPA co-infection is an independent risk factor for mortality in PCP. Consistent with previous research results, the risk factors for IPA were the previous use of tyrosine kinase inhibitors, leukopenia, and ICU admission [30,43,44]. Moreover, COVID-19 infection within 30 days was an independent risk factor for IPA co-infection [20,31,45]. Therefore, IPA co-infection should be actively evaluated in patients with PCP to improve outcomes, especially if they have a COVID-19 infection within 30 days, ICU admission, or pre-existing IPA risk factors. In the subgroup analyses of the COVID-19 era and 2022, COVID-19 was not associated with PCP-related mortality. Further studies are needed on national statistics on the incidence and outcomes of PCP after recovery from COVID-19 and the effectiveness of PCP prophylaxis in patients with COVID-19, especially in those receiving glucocorticoids and immunosuppressive drugs.

Our study has several limitations. First, this was a single-center retrospective study conducted at a tertiary university hospital. Therefore, our results may not be generalizable to other hospitals and regions of the country, and the presence of unmeasured confounding factors cannot be excluded by the retrospective nature of the study. Second, presumptive diagnoses of PCP based on negative PCR results were excluded and beta-D-glucan assays, which are used as an adjunct to the diagnosis of PCP, were not performed in our study [46,47]; therefore, the diagnosis of PCP may have been underestimated. However, *P. jirovecii* PCR had a 98.7% negative predictive value for the diagnosis of PCP [48]. Third, in assessing the patients’ COVID-19 history, we only included patients diagnosed with COVID-19 at hospitals or public health centers. Undiagnosed patients who were asymptomatic or mildly symptomatic and diagnosed cases with self-COVID-19 antigen test were excluded. In addition, a history of COVID-19 infection diagnosed earlier than 3 months was not confirmed. Therefore, the number of recent COVID-19 cases may have been underestimated. In addition, data on SARS-CoV-2 variants were not obtained; these tests were not commonly performed in hospitals or public health centers. Fourth, although the diagnostic criteria for IPA and CMV in the EORTC/MSGERC and previous studies were referred, diagnosing IPA and CMV in patients with PCP who are critically ill can be challenging due to the limited ability to safely perform histological and imaging tests. Finally, we did not analyze whether patients with underlying diseases and the use of immunosuppressive drugs that contribute to the risk of PCP increased in the study hospital. However, we only included data from the recent 7 years, and patient-years in 2022 decreased by about 15% compared to the average patient-years in the pre-COVID-19 era. This was primarily due to the lack of manpower owing to increased COVID-19 infection among medical staff; therefore, the number of patients with pre-existing PCP risk factors may not have increased.

In conclusion, in the COVID-19 era, the incidence of PCP increased significantly, and IPA co-infection in patients with PCP also increased. IPA co-infection and previous glucocorticoid use have been identified as risk factors for PCP-related mortality. Physicians should actively evaluate and treat patients with suspected PCP and determine whether they are at risk of IPA infection. Further studies are needed, based on national statistics, on the incidence and outcomes of PCP after recovery from COVID-19 and the effectiveness of PCP prophylaxis in patients with COVID-19. 

## Figures and Tables

**Table 1:** The incidence and characteristics of patients with *P. jirovecii* pneumonia in the pre-COVID-19 era and the COVID-19 era.

Characteristics	Overall (n = 113)	Pre-COVID-19 Era (n = 42)	COVID-19 Era (n = 71)	*p*
Prevalence (/100,000 patients)	45.0	27.6	72.7	0.012
Incidence (/1000 patient-years)	22.1	13.1	37.0	<0.001
Age, years	69 (60–75)	62 (54–73)	72 (69–76)	0.002
Male sex	65 (57.5)	23 (54.8)	42 (59.2)	0.648
Underlying disease				
Connective tissue diseases	23 (20.4)	9 (21.4)	14 (19.7)	0.827
Solid cancer	43 (38.1)	19 (45.2)	24 (33.8)	0.226
Hematologic malignancy	21 (18.6)	5 (11.9)	16 (22.5)	0.160
Hematopoietic stem cell transplantation	5 (4.4)	3 (7.1)	2 (2.8)	0.359
Solid organ transplantation	6 (5.3)	2 (4.8)	4 (5.6)	1.000
Immunosuppressive drugs				
Glucocorticoids	59 (52.2)	24 (57.1)	35 (49.3)	0.420
Dose mg/day *	5 (5–10)	5 (5–30)	5 (5–10)	0.114
Prednisolone ≥ 20 mg	12 (10.6)	8 (19.0)	4 (5.6)	0.025
Chemotherapy	53 (46.9)	23 (54.8)	30 (42.3)	0.198
Calcineurin Inhibitor	10 (8.8)	3 (7.1)	7 (9.9)	0.742
Methotrexate	21 (18.6)	7 (16.7)	14 (19.7)	0.687
Mycophenolate mofetil	6 (5.3)	3 (7.1)	3 (4.2)	0.669
Biologic cytokine inhibitors	23 (20.4)	8 (19.0)	15 (21.1)	0.791
Prophylactics for PCP	15 (13.3)	6 (14.3)	9 (12.7)	0.807
COVID-19 infection within 90 days	12 (10.6)	0 (0)	12 (16.9)	0.003
COVID-19 infection within 30 days	5 (4.4)	0 (0)	5 (7.0)	0.155
Shock on initial visit	26 (23.0)	12 (28.6)	14 (19.7)	0.280
Initial moderate-to-severe PCP **	88 (77.9)	32 (76.2)	56 (78.9)	0.740
ICU admission during PCP treatment	38 (33.6)	18 (42.9)	20 (28.2)	0.110
Co-infection during PCP treatment				
Cytomegalovirus pneumonia	15 (13.3)	6 (14.3)	9 (12.7)	0.807
Invasive pulmonary aspergillosis	14 (12.4)	1 (2.4)	13 (18.3)	0.013
In-hospital mortality ***	55 (49.1)	20 (47.6)	35 (50)	0.807
PCP-related mortality	50 (44.6)	19 (45.2)	31 (44.3)	0.922
Underlying disease progression	5 (4.5)	1 (2.4)	4 (5.7)	0.649

Data are presented as median (interquartile range) or number (%). These data include one patient who was transferred to another hospital during the treatment. * Dose was converted to equivalent prednisolone dose. ** a room air PaO_2_ < 70 mmHg, an alveolar–arterial oxygen gradient ≥ 35 mmHg, and/or evidence of hypoxemia (e.g., room air O_2_ saturation < 92 percent). PCP, *P. jirovecii* pneumonia; COVID-19, coronavirus disease 2019; ICU, intensive care unit. *** These data exclude one patient whose treatment outcome was unknown.

**Table 2:** Characteristics of survivors and non-survivors in patients with *P. jirovecii* pneumonia.

Characteristics	Survivor (n = 62)	Non-Survivor (n = 50)	*p*
Age, years	74 (63–76)	69 (65–74)	0.128
Male sex	35 (56.5)	30 (60.0)	0.705
Prophylactics for PCP	6 (9.7)	9 (18.0)	0.199
Immunosuppressive drugs			
Glucocorticoids	26 (41.9)	32 (64.0)	0.020
Dose mg/day *	5 (4–8)	5 (5–20)	0.186
Prednisolone ≥20 mg	5 (8.1)	7 (14.0)	0.313
Chemotherapy	31 (50.0)	22 (44.0)	0.527
Calcineurin Inhibitor	6 (9.7)	3 (6.0)	0.729
Methotrexate	9 (14.5)	11 (22.0)	0.304
Mycophenolate mofetil	4 (6.5)	2 (4.0)	0.690
Biologic cytokine inhibitors	16 (25.8)	7 (14.0)	0.124
COVID-19 infection within 90 days	9 (14.5)	2 (4.0)	0.108
COVID-19 infection within 30 days	2 (3.2)	2 (4.0)	1.000
Blood examination at diagnosis of PCP			
WBC/μL	13,345 (11,000–14,970)	11,540 (5985–16,630)	0.086
Lymphocyte count/μL	916 (598–1373)	787 (500–1089)	0.212
Platelet, ×10^3^/μL	223 (154–245)	166 (77–247)	0.144
Albumin, g/dL	2.8 (2.6–2.9)	2.9 (2.7–3.3)	0.256
Lactate dehydrogenase, U/L	485 (366–556)	618 (407–366)	0.005
CRP mg/dL	25.1 (20.5–32.7)	10.8 (6.1–19.1)	0.233
Shock on initial visit	12 (19.4)	13 (26.0)	0.401
Initial moderate-to-severe PCP **	41 (66.1)	46 (92.0)	0.001
ICU admission during PCP treatment	11 (17.7)	27 (54.0)	<0.001
APACH II score	19 (11–26)	18 (13–22)	0.446
Co-infection during PCP treatment			
Cytomegalovirus pneumonia	3 (4.8)	12 (24.0)	0.003
Invasive pulmonary aspergillosis	4 (6.5)	10 (20.0)	0.031
Ventilator	9 (14.5)	25 (50.0)	<0.001
Invasive pulmonary drainage	4 (6.5)	11 (22.0)	0.016
Combined acute kidney injury	10 (16.1)	26 (52.0)	<0.001
Pneumothorax occurrence	2 (3.2)	5 (10.0)	0.239

Data are presented as median (interquartile range) or number (%). These data exclude one patient whose treatment outcome was unknown, owing to transfer to another hospital during treatment. * Dose was converted to equivalent prednisolone dose. ** A room air PaO_2_ < 70 mmHg, an alveolar–arterial oxygen gradient ≥ 35 mmHg, and/or evidence of hypoxemia (e.g., room air O_2_ saturation < 92 percent). PCP, *P. jirovecii* pneumonia; COVID-19, coronavirus disease 2019; ICU, intensive care unit; APACH, acute physiology and chronic health examination.

**Table 3:** Risk factors of *P. jirovecii* pneumonia-related mortality.

Risk Factors	OR (95% CI)	*p*	Adjusted OR (95% CI)	*p*
Previous glucocorticoids use	2.5 (1.1–5.3)	0.021	3.0 (1.1–8.1)	0.029
Initial moderate-to-severe PCP *	5.9 (1.9–18.6)	0.002	7.9 (1.8–34.3)	0.006
ICU admission	5.4 (2.3–12.8)	<0.001		
Ventilator	5.9 (2.4–14.5)	<0.001		
Invasive pulmonary drainage	4.1 (1.2–13.8)	0.023	4.4 (0.9–22.4)	0.077
Acute kidney injury	5.6 (2.3–13.5)	<0.001	6.0 (2.1–16.8)	0.001
CMV co-infection	6.2 (1.6–23.5)	0.007	3.3 (0.8–14.3)	0.102
IPA co-infection	3.6 (1.1–12.4)	0.040	7.2 (1.4–36.8)	0.019
Lactate dehydrogenase ≥500, U/L	2.9 (1.3–6.3)	0.008		
CRP ≥ 5, mg/dL	2.7 (1.1–6.8)	0.035		

* Room air PaO_2_ < 70 mmHg, alveolar–arterial oxygen gradient ≥ 35 mmHg, and/or evidence of hypoxemia (e.g., room air O_2_ saturation < 92 percent)). OR, odds ratio; CI, confidence interval; PCP, *P. jirovecii* pneumonia; ICU, intensive care unit; CMV, cytomegalovirus; IPA, invasive pulmonary aspergillosis.

**Table 4:** Risk factors for co-infection with invasive pulmonary aspergillosis in patients with *P. jirovecii* pneumonia.

Risk Factors	OR (95% CI)	*p*	Adjusted OR (95% CI)	*p*
Previous use of tyrosine kinase inhibitor	16.2 (1.4–191.9)	0.027	36.7 (2.4–568.9)	0.010
COVID-19 infection within 30 days	8.0 (1.0–62.1)	0.047	19.4 (1.9–198.6)	0.013
Initial leucopenia (WBC < 1000/μL)	16.2 (1.4–191.9)	0.027	19.5 (1.3–296.9)	0.032
ICU admission	4.3 (1.3–13.9)	0.015	5.9 (1.4–24.6)	0.016
Ventilator	3.7 (1.2–11.7)	0.026		

OR, odds ratio; CI, confidence interval; COVID-19, coronavirus disease 2019; ICU, intensive care unit.

## Data Availability

Data presented in this study are available upon request from the corresponding author. These data are not publicly available because of privacy restrictions.

## Supplementary Materials

The following supporting information can be downloaded at: https://www.mdpi.com/article/10.3390/life13061335/s1. Table S1: Characteristics of survivors and non-survivors in patients with *P. jirovecii* pneumonia.
