# Supplementary material for: Changing Trends in the Incidence and Clinical Features of Pneumocystis jirovecii Pneumonia in Non-HIV Patients before and during the COVID-19 Era and Risk Factors for Mortality between 2016 and 2022
Source: Life (Basel). 2023 Jun 7;13(6):1335. doi: 10.3390/life13061335 (PMC10304474; doi:10.3390/life13061335)
Supplement: Supplementary file 1 [file life-13-01335-s001.zip › life-2415970-supplementary.pdf]

**Supplement Materials.**

**Supplementary Table S1. Characteristics of survivors and non-survivors in patients with *P. jirovecii* pneumonia**

| Characteristics | Over all (n=112) | Survivor (n =62) | Non-survivor (n =50) | *p* |
|---|---|---|---|---|
| Comorbidity or underlying disease | | | | |
| Cardiovascular disease | 14 (12.5) | 10 (16.1) | 4 (8.0) | 0.196 |
| Cerebrovascular disease | 11 (9.8) | 8 (12.9) | 3 (6.0) | 0.340 |
| Chronic kidney disease | 20 (17.9) | 12 (19.4) | 8 (16.0) | 0.645 |
| Chronic lung disease | 18 (16.1) | 7 (11.3) | 11 (22.0) | 0.125 |
| Diabetes | 37 (33.0) | 20 (32.3) | 17 (34.0) | 0.846 |
| Heart failure | 12 (10.7) | 7 (11.3) | 5 (10.0) | 0.826 |
| Hypertension | 52 (46.4) | 30 (48.4) | 22 (44.0) | 0.644 |
| Liver disease | 17 (15.2) | 8 (12.9) | 9 (18.0) | 0.455 |
| Connective tissue diseases | 22 (19.6) | 11 (17.7) | 11 (22.0) | 0.573 |
| Solid cancer | 43 (38.4) | 26 (41.9) | 17 (34.0) | 0.391 |
| Hematologic malignancy | 21 (18.8) | 13 (21.0) | 8 (16.0) | 0.503 |
| Hematopoietic stem cell transplantation | 5 (4.5) | 2 (3.2) | 3 (6.0) | 0.655 |
| Solid organ transplantation | 6 (5.4) | 5 (8.1) | 1 (2.0) | 0.222 |
| Charlson Comorbidity Index score | 5 (4-8) | 5 (3-7) | 5 (3-6) | 0.436 |

Data are presented as median (interquartile range) or number (%).

These data exclude one patient whose treatment outcome was unknown due to transfer to another hospital during treatment.
